# Ultrafast-Contactless Flash Sintering using Plasma Electrodes

**DOI:** 10.1038/srep27222

**Published:** 2016-06-08

**Authors:** Theo Saunders, Salvatore Grasso, Michael J. Reece

**Affiliations:** 1 School of Engineering and Material Science, Queen Mary University of London, London, E1 4NS, UK

## Abstract

This paper presents a novel derivative of flash sintering, in which contactless flash sintering (CFS) is achieved using plasma electrodes. In this setup, electrical contact with the sample to be sintered is made by two arc plasma electrodes, one on either side, allowing current to pass through the sample. This opens up the possibility of continuous throughput flash sintering. Preheating, a usual precondition for flash sintering, is provided by the arc electrodes which heat the sample to 1400 °C. The best results were produced with pre-compacted samples (bars 1.8 mm thick) of pure B_4_C (discharge time 2s, current 4A) and SiC:B_4_C 50 wt% (3s at 6A), which were fully consolidated under a heating rate approaching 20000 °C/min. For the composite a cylindrical volume of 14 mm^3^ was sintered to full density with limited grain growth.

In ~24,000 BC ceramics were produced by firing clay objects in furnaces heated by burning wood^1^. This process resulted in the consolidation of the material, and was time and energy intensive. In conventional sintering, the heating is not directed only to the parts to be sintered but also to the furnace (refractories and heating elements) and a great part of it is also irreversibly wasted (heat exchanges with the surroundings). Apart from technological improvement of heating furnaces, the sintering process has remained unchanged for many centuries. Recently, the use of heat generated by current flowing directly through the sintering material (as in a heating element), has been proposed as a more energy efficient way of consolidating particles. As opposed to conventional sintering techniques, electrical heating is localized to the sintering material, thus, minimizing the fraction of wasted heat.

Flash Sintering (FS) refers to the sudden densification observed when high fields (60–100 V/cm) are applied across a sample (e.g. yttria stabilized zirconia) pre-heated to a suitable temperature (≈850 °C). Similar FS behaviour has been found in a variety of ceramics^2^,^3^,^4^,^5^,^6^,^7^,^8^. The exact mechanism has not been proven conclusively and there is much academic debate on the subject, however, several researchers have focused on Joule heating causing thermal runaway^9^,^10^. Flash sintering offers many potential advantages over conventional sintering in energy efficiency, production of metastable microstructures/compositions and production rate. However, there are many complications that flash sintering also brings, which limit its commercial application. The need to have electrodes attached to the surface of the sample is a challenging issue. The initial choice of electrodes was platinum^3^ with obvious cost issues. The use of electrodes adds several extra steps to the FS process, since conductive electrodes have to be attached and then removed after sintering. The requirement for electrodes also limits the geometry of sintered parts. A uniform electric field and current flow is required to achieve homogeneous heating. This means complex geometries (having non-constant thickness) cannot be sintered homogenously. The development of Contactless FS (CFS) technology would not only overcome these problems but might also allow continuous sintering by passing current through a material which is in relative motion with respect to the electrodes. To the authors’ best knowledge, there are at least two ways to achieve FS in contactless mode. Microwave FS, especially in single mode cavities, is capable of rapid sintering (with a heating rate of a few thousands of degree per minute)^11^,^12^,^13^,^14^. However, the use of microwaves has limitations with hotspot size limiting either the speed of sintering or the homogeneity/size of the samples^13^.

In this paper we present a novel and flexible method for FS ceramics which employs a conductive plasma as electrodes, which also integrates the preheating of the material. In existing FS technology, preheating (to a temperature above which the electrical conductivity suddenly increases) is usually done in conventional furnaces, which greatly adds to the processing time and energy requirements, making FS less energy attractive. A plasma being composed of ions and free electrons can carry current and conform to the surface it is in contact with. The simplest design is to have two metal electrodes and the sample held between them with a gap. Two independent plasmas are generated at the electrodes as depicted in Fig. 1, and current can then flow through the sample across the plasma. However, this configuration is not practical, as each plasma electrode would require so much support equipment that two could not be placed even relatively close together.

## Development of the Experimental Setup for Contactless Flash Sintering (CFS)

There are a variety of different methods and equipment used to generate plasmas, and they can produce plasmas with very different properties^15^,^16^,^17^,^18^. For the purpose of this paper, the thermal and electrical properties of the plasma are considered. The electrical conductivity of a plasma is complex and it depends on many different environmental conditions/parameters. However, if all that is needed is a broad comparison of orders of magnitude, electrical conductivity can be considered to be proportional to the electron number density. Electron number density is the number of free electrons in the plasma per unit of volume as listed in Table 1.

**Figure 1:**
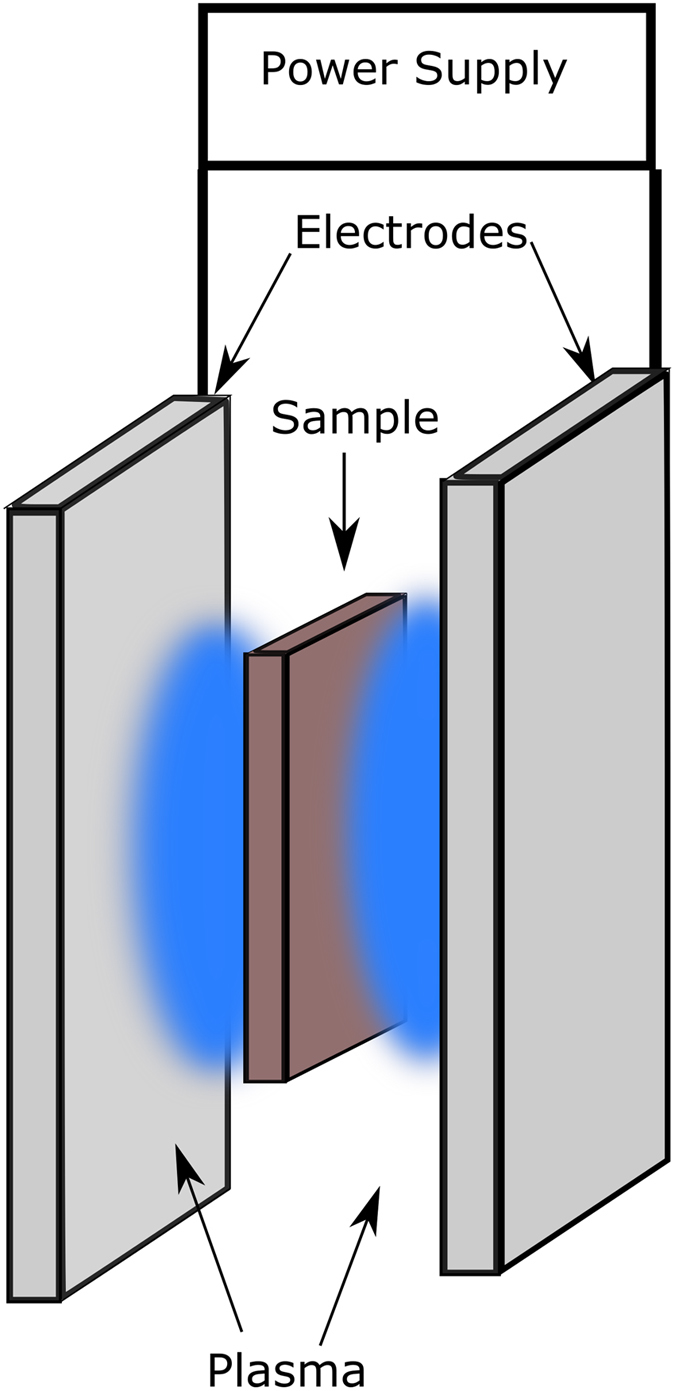
A schematic of essential components to realize contactless flash sintering based on plasma electrodes.

To allow CFS, the plasma should have a temperature suitable for preheating the sample, so a cold plasma would be preferred (low neutral temperature) for materials having low sintering temperatures (below 1000 °C). A plasma with a high electrical conductivity would result in a low voltage drop through the plasma. Looking at Fig. 1, a plasma that could homogenously cover a large surface (on both sides of the sample) would allow the entire volume of a sample between the electrodes to be sintered. A RF glow discharge or inductively coupled plasma are the most likely candidates to meet the above requirements. However, both of these methods require a completely controlled atmosphere and complex plasma generation equipment. A further complication is the presence of an electric current in the plasma, which can change drastically the proprieties of the plasma. By looking at the traditional voltage current curve for gas discharge^17^ (Fig. 1s in supplementary information), It is likely that the plasma will degenerate into an arc discharge under normal flash sintering conditions when the current exceeds 1 A. To avoid the degradation of the plasma electrodes, it is necessary for the electrode materials to be compatible with the high temperature of the arc discharge.

**Figure 2:**
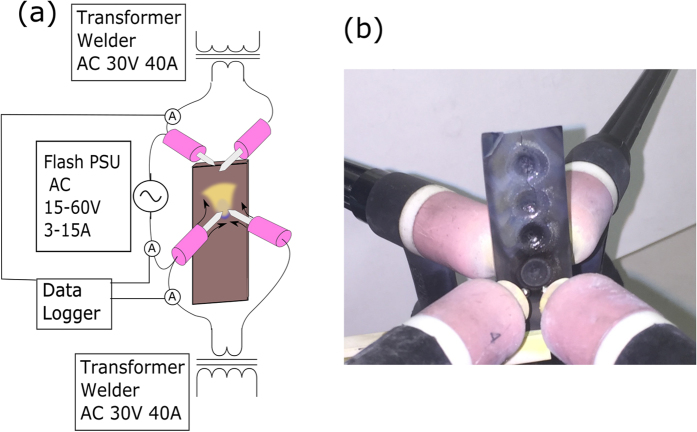
(**a**) A schematic of the plasma flash experimental setup. Two isolated transformer welders were used to generate two electric arcs, a flash power supply was used to pass current through the sample. (**b**) A photo of two twin torches and processed sample.

An advantage of an arc plasma is that it only requires simple equipment; a step down transformer with a magnetic shunt as is used in a traditional welding power supply. There is good reason to use a simple transformer style welder instead of more modern transistor systems. By using an AC welding transformer the three power supplies are effectively isolated from one another by their magnetic cores (see simplified electric schematic in Fig. 2(a)). This avoids issues where common ground faults would short out the power supplies with disastrous results. The choice of arc plasma electrodes is not without its disadvantages, the high neutral temperature (over 5000K^19^) of the arc plasma will transfer significant heat into the sample. One upside of this is that the samples will already be heated by the arc electrodes, which renders external preheating unnecessary, which further simplifies the experimental setup. AC power supplies were used because arc heating is effected by polarity; in DC operation the +Ve surface of the sample would adsorb 70% of the heat. Therefore the use of an AC power supply would allow a more even and symmetric heating compared to DC arcs.

## Method

A schematic of the experimental set up used for CFS is shown in Fig. 2(a). The experimental procedure was to load a pre-compacted sample (prepared as described in the supplementary information) into a ceramic holder. The sample was placed under the arcs using a 3 axis positioner. The two arcs were ignited in turn and the sample was then raised up so as to be in between the arcs, equidistance between them. The sample was preheated for 5s and then the flash power supply was activated with a set current limit (eg ≈6A) for a fixed period of time (eg ≈2,3,4 and 5s).The setup and geometry for CFS was optimized carefully for the materials investigated and is described in detail in the supplementary information.

Several materials were investigated, pure SiC, SiC:B_4_C and pure B_4_C. These were chosen due to their high temperature stability, so that they were not damaged by the arc and have reasonable thermal conductivity at high temperatures so that preheating can be achieved by the heating from the arc. Due to their different sintering behaviour and electrical properties, various processing conditions were used in an attempt to achieve full density.

The flash sintering current was supplied using a Super 200P 4 in 1 welder. The currents in the two plasma arcs and the flash sintering current were measured using Hall effect sensors (Allegro Microsystems, LLC ACS758LCB-100B) connected to a data logger (National Instruments USB-6221). The preheating temperature was measured in a separate experiment using a K type thermocouple 1 mm in diameter embedded in the sample.

After CFS the samples were photographed and inspected using optical microscopy (Nikon SMZ-10) to evaluate the sintered region. Following this, the samples were embedded in resin and cut across the centre of the sintered region and polished for SEM analysis. SEM micrographs were taken in secondary emission mode at 20 kV on a FEI inspect F unit.

## Results and Discussion

Before delving into the results, it is useful to clarify what happens during flash sintering. Before the flash event the arcs heat the sample from the surface so that a discrete sample volume becomes hot enough to be electrically conductive (above or near the flash sintering threshold temperature). On application of the flash power supply an arc is struck between the electrodes and the surfaces of the sample. This results in heating of the surface due to heating from the plasma and electron bombardment, but also Joule heating inside the sample. It is this fact that distinguishes this method from arc heating and melting and makes it a flash sintering process. Evidence of this can be seen in the first seconds of flash sintering and from some of the microstructures which show evidence of thermal gradients produced by Joule heating.

**Figure 3:**
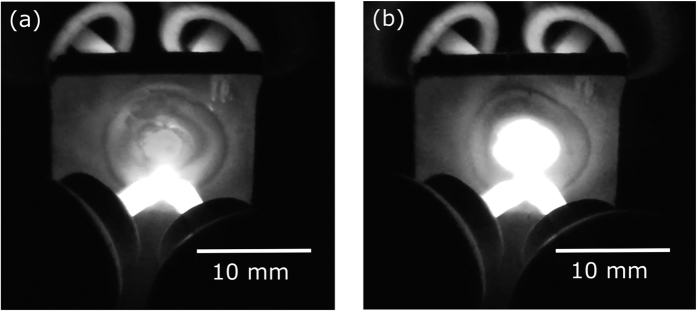
Showing stills from a video of the contactless flash sintering process, (**a**) before and (**b**) 0.5s after the application of the flash power supply reaching a current of 6 A.

The total power supplied by the welding transformer to each twin arc was ≈800 W (19 V and 43A RMS, see Fig. 2s in the supplementary information). Despite this high power, when the sample was held between the arcs for 60s no densification was observed. Arc preheating caused the temperature to rapidly rise to 1400 °C within 5s (as shown in Fig. 3s in the supplementary information). This temperature, which was lower than the pre-compaction temperature of the sample (1550 °C), was not sufficient to further densify the material. This is due to the poor thermal coupling of the (preheating) arcs with the sample. In an arc the heat is mainly carried by the electrons, so most of the heating was localized at the tungsten electrodes. The CFS power supply produced much better coupling by a combination of internal Joule heating and from electrons striking the surface of the sample. This explains the rapid heating seen in Fig. 3(b), where a ripple (4–5 mm diameter) of heat is seen spreading across the surface of the sample the instant the flash power supply (≈200 w) was switched on. It is worth noting that the developed setup produced power densities exceeding 10000 mW/mm^3^, values that are not achievable in other flash sintering configurations^2^,^3^,^4^,^5^,^6^,^7^,^8^. This allowed us to achieve instantaneous (<3s) full consolidation of covalent ceramics (B_4_C and their mixtures with SiC).

### Results for different materials

A selection of materials were processed in preliminary experiments to determine a reasonable combination of time and current for each of the materials. From that a series of structured experiments were devised. Current was found to be much more important than time, so once a good setting for current was obtained it was fixed and then various times were investigated. Below are figures showing a high mag micrograph of the best microstructure achieved, with our current optimized processing conditions, for the three materials.

**Figure 4:**
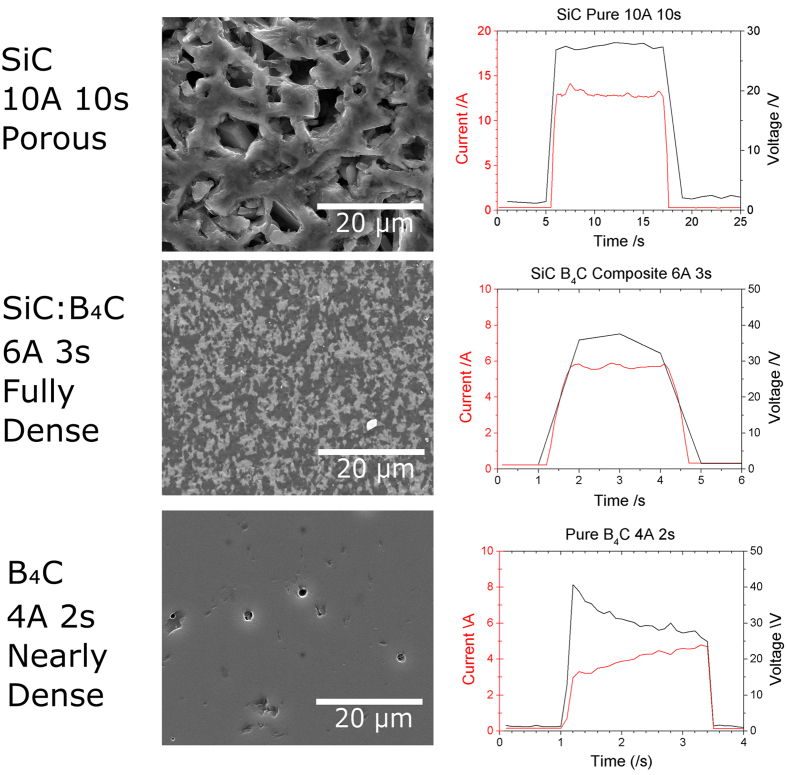
SEM micrographs of the best microstructures for each material (pure SiC, SiC:B_4_C 50 wt% and pure B_4_C) and the electrical data (current and voltage) from the representative CFS runs.

As can be seen in Fig. 4, the composite (SiC:B_4_C 50 wt%) and pure B_4_C had dense microstructures after CFS. The pure SiC had a porous, recrystallized microstructure, which is typical for pure SiC. It was not possible to achieve dense materials regardless of the applied currents and discharge times. This microstructure originates because of the sublimation (at temperatures above 2000 °C) and condensation of SiC within closed pores. The composite (SiC:B_4_C 50 wt%) was evenly sintered and had a homogenous microstructure. In this study, the initial average particle size of the starting powder was 0.7 and 0.5 μm for SiC and B_4_C respectively (according to manufacturer’s specification). For the sample CFSed for 3s the grain sizes, were 1.1 μm for SiC and 1.4 μm for B_4_C. Because of these promising results a more thorough investigation was undertaken to understand the sintering behaviour of the composite (SiC:B_4_C 50 wt%). Samples were CFSed at various times, and cross sectioned, providing snapshots of the process as it progressed. A lower current was also investigated to understand the effect of current on the sample, both at the micro and macro scale. This investigation is documented in detail in the supplementary information.

### Electrical Data

The voltage and current were logged during the sintering process and various features are noticeable in the data. It is important to note that this is the data from the whole system, so the voltage recorded is the voltage between the tungsten electrodes and not simply across the sample. Due to the highly nonlinear resistance of the arcs, as detailed in the supplementary information, the voltage drop across that arcs cannot be easily estimated, so cannot be subtracted to find the true sample voltage.

An interesting feature of flash sintering relates to the preheating and incubation. From the electrical data in the supplementary information, only the pure B_4_C samples under certain conditions showed an incubation period. This is hypothesized to be due to the slope of resistivity vs temperature curve for B_4_C being relatively constant^20^. In contrast to SiC, which tends to show a sudden drop in resistance^21^ when the semiconductor reaches its intrinsic region at temperatures exceeding 1400 °C.

Due to the experimental setup, perhaps the only experimental parameter that can be used as a term of comparison is the current density. This was calculated by taking the diameter of the sintered region and the flash sintering power and was 0.6 A/mm^2^ for pure SiC (recrystallized microstructure), 0.3 A/mm^2^ for SiC B_4_C (fully dense) and 0.32 A/mm^2^ for pure B_4_C (nearly full dense).

## Conclusion

This work demonstrates for the first time contactless flash sintering. The electric current required to flash sinter the material was carried through a plasma, thus avoiding the limitations/difficulties imposed by the use of electrical contacts (i.e. contamination of the contact region and unnecessary extra processing steps). A plasma being composed of ions and free electrons can carry current, and will conform to the surface of the material being sintered, thus allowing flash sintering without the need for electrodes in physical contact with the sample. The absence of contacts provides great versatility to the FS process, making it possible to achieve continuous sintering, where the sample is set in relative motion with respect to plasma electrodes. The proposed methodology can be applied to flash sinter a wide range of materials. Guidance for the selection of suitable plasma type are developed in the paper (plasma temperatures, plasma conductivities and maximum currents). Notably, starting from a SiC 50%wt B_4_C block with initial relative density of 65%, contactless flash sintering produced a fully dense cylindrical region with a volume of 14 mm^3^ in a discharge time of 3s with a current of 6A. The roughly estimated heating rate was in the order of 14000–18000 °C/min. The microstructure was found to be very dependent on the discharge time.

## Additional Information

**How to cite this article**: Saunders, T. *et al.* Ultrafast-Contactless Flash Sintering using Plasma Electrodes. *Sci. Rep.* **6**, 27222; doi: 10.1038/srep27222 (2016).

## Supplementary Material

Supplementary Information

Supplementary Video S1

## Figures and Tables

**Table 1:** A summary of different plasma generation techniques and the properties of the plasma that they produce.

Plasma Source	Electron number density/m^−3^	Background pressure/Torr	nominal power^*^ /W	Reference
DC glow discharge	10^16^	0.10–5	100–300	16
RF glow discharge	10^17^	0.05–1	200–500	16
Electron cyclotron resonance	10^18^	10^−4^–0.01	300–1,000	16
Inductively coupled	10^18^	10^−3^–0.1	500–2,000	16
Helicon	10^18^–10^19^	0.01–0.1	500–2,000	16
DC plasma jet	10^20^–10^23^	750–75,000	1,000–20,000	22
DC welding arc	10^19^–10^23^	750	500–2,000	23
