# Supplementary material for: Ultrafast-Contactless Flash Sintering using Plasma Electrodes
Source: Sci Rep. 2016 Jun 8;6:27222. doi: 10.1038/srep27222 (PMC4897693; doi:10.1038/srep27222)
Supplement: Supplementary Information [file srep27222-s1.pdf]

Ultrafast-Contactless Flash Sintering using Plasma Electrodes

Theo Saunders[a], Salvatore Grasso[a*], Michael J Reece[a]

[a]School of Engineering and Material Science, Queen Mary University of London, London, E1 4NS, UK

Supplementary Information

Intro

For the sake of brevity in the main paper various details required for duplication of the CFS (contactless flash sintering) process are included here instead. While the paper serves as a proof of concept, results from various investigations into the process are also included here for those who would like a more in depth understanding of the process.

The method in detail

The main decision to use an arc power supply was driven by the simplicity of the equipment but also the graph in Figure. 1s that shows that any other type of plasma electrode would rapidly degenerate into an arc.

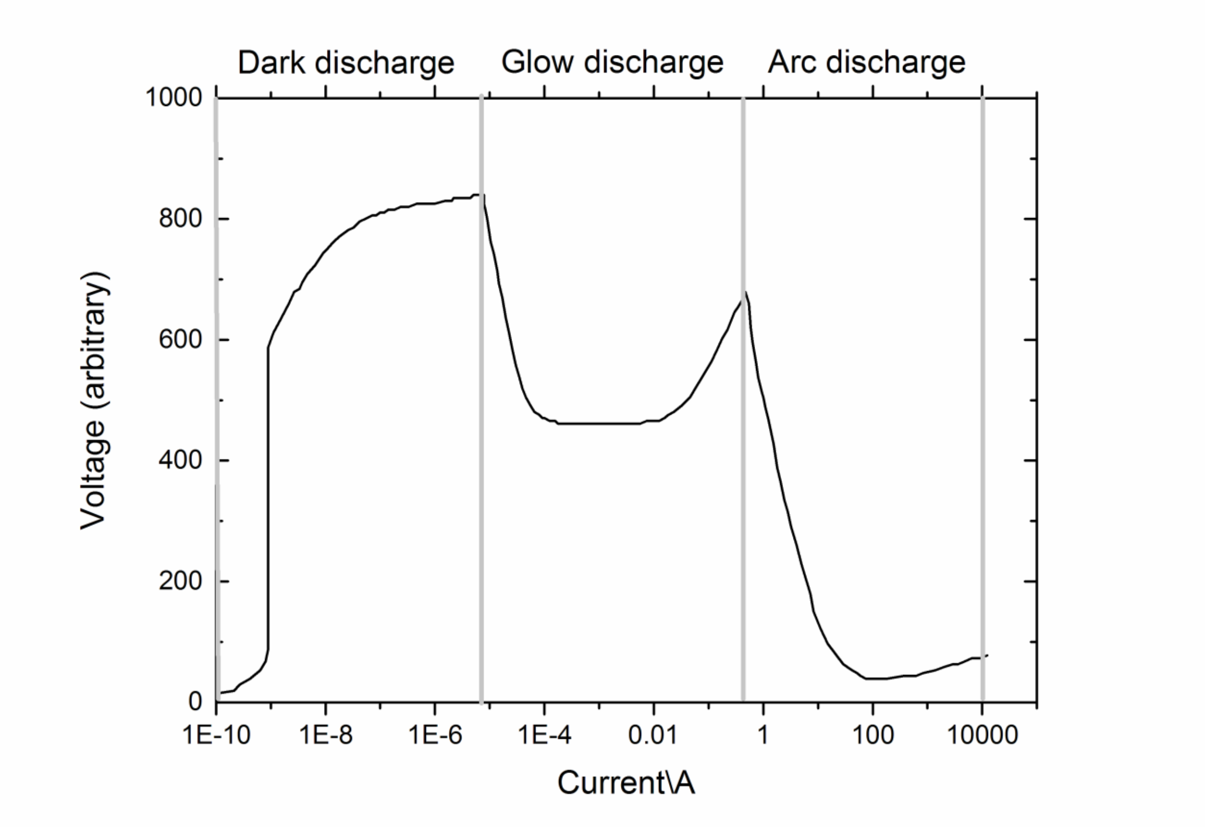

Figure. 1s. I-V curve for welding arcs at similar conditions to those used in flash sintering, adapted from figures from The welding institute's online resources [1].

While the process is summarised in the main paper, to achieve a reliable process various processing parameters needed to be optimised. These details are described below.

Samples were prepared using SPS (FCT HD) equipment to produce porous discs that were then cut into 1.8mm slices. Thin slices (18 mm by 1.8mm by ≈40mm) were cut using a diamond disc. After cutting, the samples were dried in an oven prior to flash sintering as moisture caused cracking under the preheating by the arc.

The materials used and the sintering conditions are summarized in Table 1s below. It is worth noting that the samples were sintered only so that they had sufficient mechanical strength to be cut to shape. Potentially a cold pressing technique could be used instead to reduce energy, time and cost.

Table 1s. A summary of the different processing conditions for the materials used.

**Material**	**Grade**	**Sintering maximum temp /°C**	**Sintering pressure /MPa**	**Sintering hold time /min**	**Relative density /%**
**SiC**	Starck Grade UF 10	2000°C	10	5	67
**$SiC:B_4C$**	Mix of both roller milled in ethanol 18hr	1550°C	40	30	65
**B_4C**	Starck Grade HD 20	1600°C	20	5	70

The optimal setting and layout for the plasma torches was chosen after exhaustive preliminary testing. The AC transformer welder's current was set to 40A to control sample heating and wear of the tungsten electrodes. The arc gap (distance between the two tungsten rods in each pair of TIG torches) was chosen to produce a well-defined two zone plasma plume without being too large to destabilize the arc; 1-2mm was found to be a good range only needing occasional adjustment as the tungsten rods wore down. The argon flow rate (1L/min) and cup size (no4 gas lens, diameter 6.35mm) were adjusted to provide sufficient coverage to avoid oxidation of the tungsten rods while still allowing easy ignition of both pairs of torches. Under these conditions and given the resulting AC waveform, the calculated power is given in Figure. 2s below.

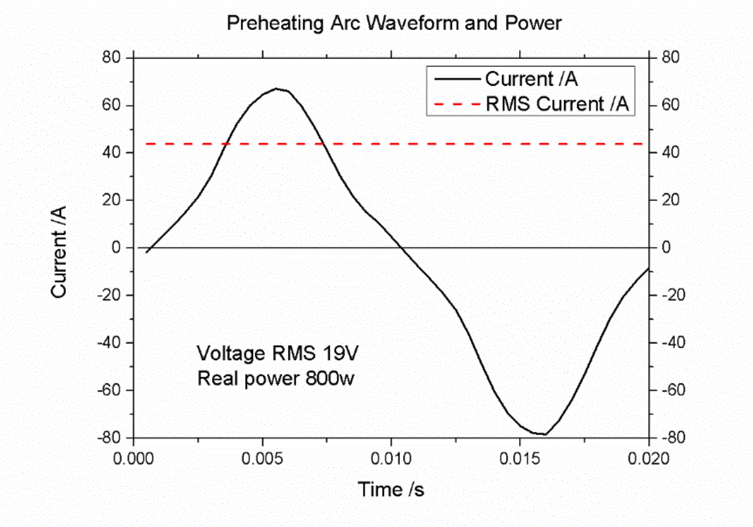

Figure 2s. A plot of the AC waveform of the transformer welder. The root mean square (RMS) value of current is included for comparison. Combined with the RMS voltage, measured from the isolated multi meter, the real power was calculated.

For the arc ignition, an arc was struck between the two tungsten rods. This was achieved by shorting them out with a carbon rod and then breaking the contact (this is based

on a common procedure in TIG welding called scratch start). Once both pairs of torches were lit, the sample was inserted between them. The distance between the two pairs of torches was critical; if the separation was too great then there was insufficient preheating as the hot spot in the sample did not penetrate the entire thickness of the sample. This also explains the importance of the thickness of the sample (1.8-2mm). The separation was kept constant at 14mm for all experiments. Thicker samples did not allow the heating to penetrate all the way through the sample. An attempt was made at quantifying the preheating temperature reached, and this is plotted in Figure 3s. For this test the sample was correctly positioned between the torches (the sample had already reached 400°C at this point), and once in position the temperature rapidly rose to 1400°C in just 5s. For this reason the sample was preheated for 10s in the actual experiments to ensure an even and repeatable preheat.

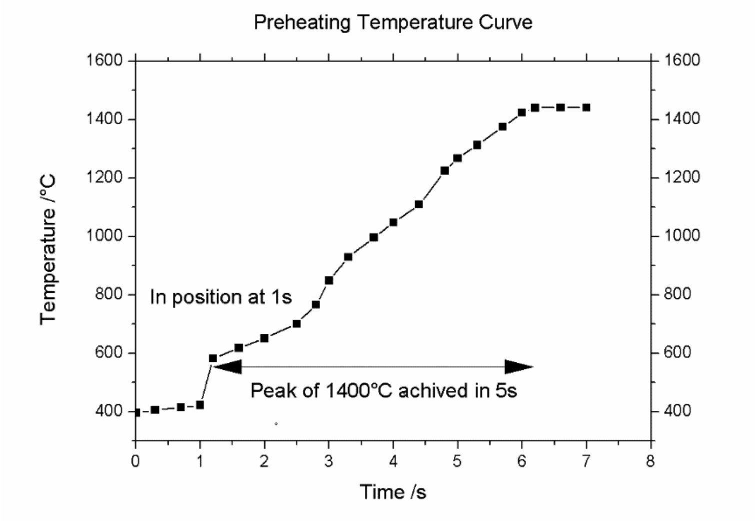

Fig. 3s. Time temperature preheating curve measured using an embedded thermocouple.

The sample was raised into position so the centre of the hotspot was over the thermocouple. By the time both arcs were lit and the sample was in position, the temperature was already 450°C, and this was used as the 1s mark. The same size (18x1.8x50mm^3) and composition (SiC:B_4C 50:50wt%) samples were used as for the CFS experiments. It is worth noting that while these are the setting that worked for our experiment, various other setting would have likely worked. If attempting scale up of the process various other factors would come into play. In fact the process should become more stable with increasing currents and sizes as larger arcs are more stable. More focused plasma sources such as plasma arc welding torches could potentially increase the process. A video showing the contactless flash sintering process for SiC:B_4C 50 wt% processed under 6 A is available.

Video link

(caption) Video showing the CFS densification of SiC:B_4C 50 wt% processed under 6 A for 3 s. Flash sintering initiates when a bright light appears and current (≈0.3A/mm^2) starts flowing across the sample.

Results

With the methodology explained in detail we can investigate how the flash sintering process progresses. Before that it is useful to understand what happens in the whole sample, not just a small region. Figure 4s shows a representative example of a sample directly after CFS. As can be seen, only a small region of the sample was affected and a crater like structure formed where the material in the centre densified. The surface, however, was not representative of the underlying material, so the samples were cross sectioned about the centre of the crater. A schematic of the different zones typically seen is shown in figure 5s.

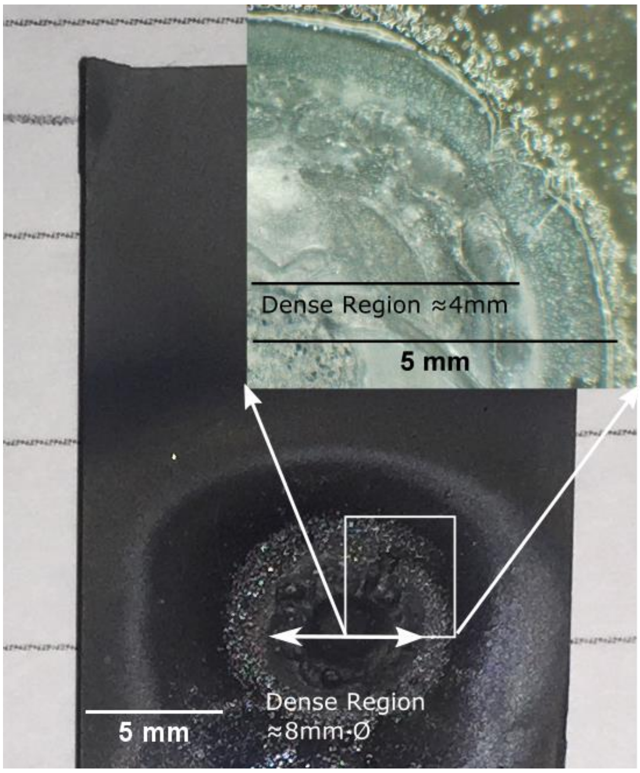

Fig 4s. Photograph of the samples CFSed for 2s and inset showing optical micrograph highlighting surface modification of the SiC 50 wt% B_4C induced during the processing.

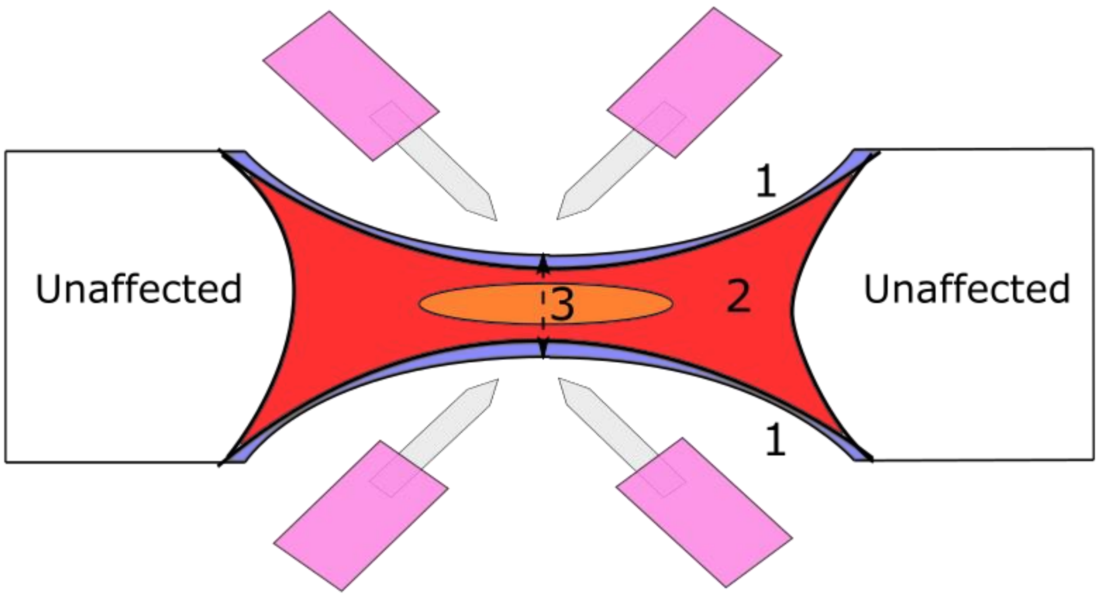

Fig 5s. A schematic of a typical CFS sample detailing the location and relative size of the resulting Zones (described in the text).

Composite SiC:B_4C

This section will focus on the processing of the SiC:B_4C composite at 6A as it produced the best microstructure. As seen in Figure 4s, the surface of the samples was modified by the CFS process. The inset of Figure 4s shows a carter impression (due to the reduced thickness from sintering) having a diameter of 10mm. Inside this region only an inner portion of 5 mm diameter is significantly thinner/denser, in agreement with the hot spot size in Figure 3 (main paper). The surface in contact with the plasma was modified by electron bombardment which resulted in an excessive local heating. The depth of this layer was limited as discussed below. The discoloration located outside the crater (visible in inset of Figure 4s) was produced by a thin oxide layer formed when the sample cooled outside of the argon flow.

Depending on the processing time, up to three different Zones (Figure 5s) were visible in the microstructure. A consistent finding was the higher temperature on the surface of the material (in contact with the plasma) compared to its mid thickness. The region near the surface of the arc crater showed melting and recrystallization (Zone 1). Further inwards a dense fine grained structure was formed (Zone 2). At the sample mid thickness, under or overs sintering was often visible (Zone 3). Depending on the CFS time, the transition between the different zones was more or less marked. Table 2s was derived from measurements made on the samples shown in Figures 6s (a-d). The 2s and 3s samples were very similar, the only major difference was the porosity in the middle (zone 3), which was only visible at high magnification (which is why a high magnification micrograph of this zone is shown). As seen In the Figures 7, the samples showed cracking induced by the differential shrinkage during CFS processing. This issue could be solved by sintering large portions of material or using a more uniform preheating. As summarized in Table 2s, the initial thickness of the sample was

1800-2000 µm, and after 2,3,4 and 5s of CFS the thickness was 1200,1100,700 and 1100 µm respectively.

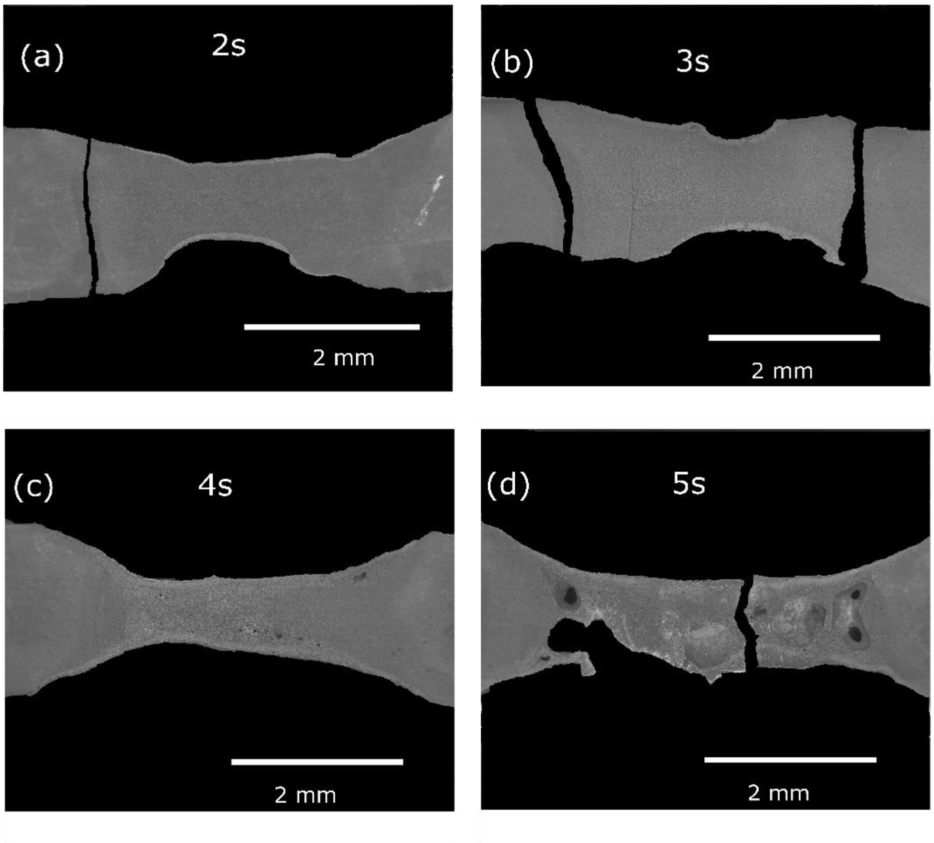

Figure 6s. Micrographs of plasma flash processed SiC:B_4C showing the progression of sintering over CFS discharge time; (a) 2s; (b) 3s; (c) 4s; and (d) 5s.

From Figure 6s and Table 2s it is possible to piece together the sequence of events that occurred as the samples were CFS. First, the sample was preheated up to 1400°C (see Figure 3s in supplementary material) by the arc torches, which produced a diffuse heating over a region of ~10 mm in diameter (Figure 3 main paper). When the CFS power supply was switched on it generated an arc against the surface of the material. As the arc was transferred to the sample, the surface was bombarded by electrons and ions, causing rapid heating of the surface. As seen in Figure 3 (a) and (b) the hot spot rapidly expanded within 0.5s to 5mm in diameter, melting a thin layer of material (Zone 1). The temperatures reached in Zone 1 must have exceeded the eutectic temperature of SiC-B_4C which is 2300°C [2].

.

**Time /s**	**Zone 1**<br>**Surface**	**Zone 2**<br>**Interior**	**Zone 3**<br>**Centre**
**2s Microstructure**	melted	sintered porous	sintered highly porous
**Thickness (900µm total)**	80µm	170µm	400µm

**3s Microstructure**	melted	fully sintered (900 µm)
**Thickness (1100µm total)**	100µm	Zone 2 and 3 indistinct
**4s Microstructure**	melted	sintered bubble (380 µm)
**Thickness (700µm total)**	160 µm	Zone 2 and 3 indistinct
**5s Microstructure**	melted and bubbles (1,100 µm)	
**Thickness (1100µm total)**	Zone 1-3 are indistinct	

Table 2s. Summary of microstructures observed in the different Zones of the sample processed by CFS after 2,3,4 and 5s. The layer thickness and diameter (Φ) of the different Zones are also given. Initial sample thickness 1.8 mm

Two seconds after the initial arc transfer the surface heating had diffused deeper into the sample, which caused the melted Zone 1 to grow to 80µm thick. Material near Zone 1 began to sinter, forming Zone 2, with a thickness of 170µm. Zone 3 (400µm thick), was still relatively cool and so remained highly porous. The sample was about 1200µm thick at its most narrow section.

Given even more time (3s) the melted layer (Zone 1) grew thicker (100µm), consuming more of the sintered region (Zones 2). Zones 2 and 3 were now indistinguishable (labelled as zones 2/3 in Figures 7s as both were dense with no bubbles or abnormal grain growth, with a thickness of 900µm.The sample was now 1100µm thick at its most narrow section. The theoretical thickness predicted for fully dense material (starting from 1800 µm (65% dense)) is 1170 µm, so the narrow deviation from the measured value suggests that almost no material was lost during CFS.

Given even more time (4s) the molten layer (Zone 1) expanded to 160µm and the sample started ablating and thinning, and the total thickness was now 700µm). Hotspots formed inside the sample at the boundary between Zones 1 and 2/3. These regions overheated to the point where SiC started to sublime, forming bubbles as the gas was trapped by the molten Zone 1. This also indicated that the temperature inside the sample had reached at least 2200-2400°C, the point at which SiC sublimes rapidly [3]. In Zone 2/3 (now 380µm thick) the temperature continued to rise which caused significant grain growth.

Given even more time (5s) the entire sample melted, and platelets formed by precipitation form the liquid phase and bubbles formed throughout. This actually caused the sample to expand to 1100µm thick, even as material was being lost by sublimation/ablation

Because of its more homogenous microstructure, the sample CFSed for 3s is described in more detail. From the low magnification micrographs (Figure 6s (b)) it was evident that Zones 2 and 3 were indistinct. Figures 7s shows the microstructure in more detail for each of the Zones. Zones 2/3 were nearly fully dense with no pores visible even at 10,000X magnification. Consolidation occurred over a sizeable region of the flash sintered sample with a volume (derived from SEM analysis supposing cylindrical shape) of 14mm^3 corresponding to a thickness of 1100 µm and diameter of 4000 µm. Only a thin layer of overheated material

(Zone 1), showed signs of having melting, and this was limited to just the surface (≈100μm thick).

The microstructure visible in Figure 7s shows many interesting features. In Zone 2/3, the phase volume fraction measured by SEM phase analysis was 61% B_4C and 39% SiC, which is close to that expected of a 50wt% SiC:B_4C mixture (57 vol% B_4C : 43 vol% SiC). This confirmed that the formation of molten phases on the surface of the sample, visible as zone 1 in Figure 7s (b), did not significantly affect the bulk composition as measured in zone 2/3 from Figure 7s (d). In the higher of magnification image of zone 2/3 in Figures 8 (d) it is possible to clearly see the two phases and how were distributed. B_4C (dark phase) formed a continuous network matrix with the SiC (light grey phase) forming the secondary distributed phase. The phases were not entirely segregated; several individual isolated grains of SiC were visible within the B_4C matrix. The grains were multifaceted and the grain boundaries remained well defined with a marked sharp compositional gradient between the two phases. Similarly, sub-micrometre SiC grains were present in the matrix. The sharp small grains confirm the that material was a metastable phase and would have suffered increased segregation if it had been held at high temperature for longer, eventually becoming more like materials processed in more traditional conditions, such as those reported by Lankau et al[4]. They produced SiC - 40% volume B_4C composites with 98% relative density by sintering the material in argon at 2150 ^{o}C for 75 minutes. The resulting SiC grain size ranged from 2 to 5 μm. In this study, the initial average particle size of the starting powder was 0.7 and 0.5 μm for SiC and B_4C respectively (according to manufacturer's specification). For the sample CFSed for 3s the grain sizes, estimated using a method developed by Wurst and Nelson[5], were 1.1μm for SiC and 1.4μm for B_4C (Figure 7s (d)). The reduced grain growth of the SiC compared to B_4C can be attributed to the constraining grain growth effect of the matrix. In comparison with pressureless sintered samples[4] the finer SiC grain size of samples CFSed for 3s (Zone 2/3) can be explained by the very short processing time.

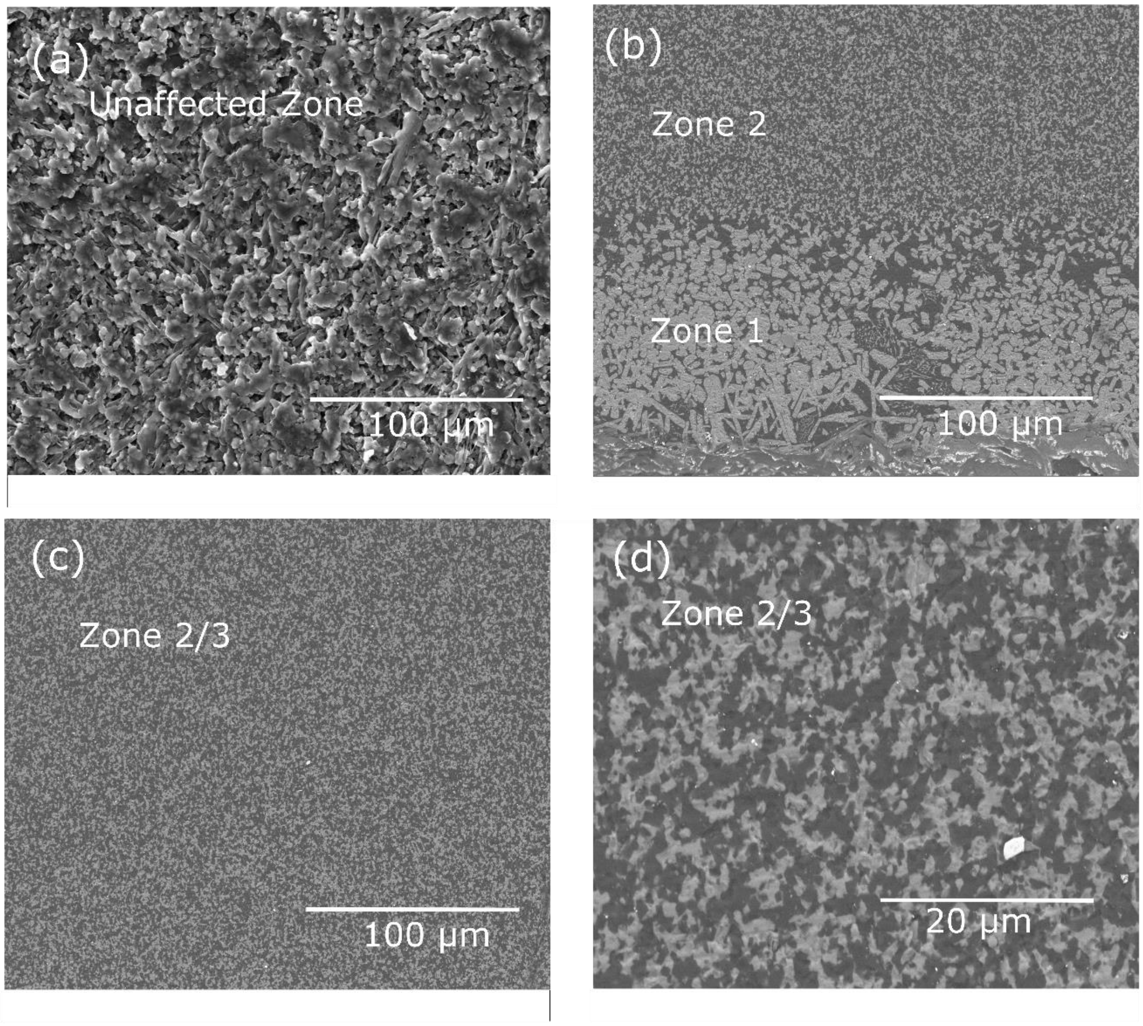

Fig 7s SEM images of SiC 50 wt% B_4C sample CFSed for 3s: (a) unaffected Zone; (b) boundary region between Zone 1 and 2/3 (the Zones were indistinct); and (c) (d) higher magnification of Zone 2/3.

Electrical data

Electrical data from the samples processed by CFS for 2,3,4 and 5 s are shown in Figures 8s (a-d). For all of the samples the arcs were stable (i.e. no current oscillations or cut offs) and there was no incubation time as when the voltage was applied the current suddenly increased. The lack of an incubation period implies that the preheating provided by the arc electrodes was sufficient to heat the sample above the thermal runaway temperature. By looking at Figure 8s (c), the electrical resistance increased with the time/temperature, this might be due to reduced conductivity of the SiC/B_4C composite at high temperature.

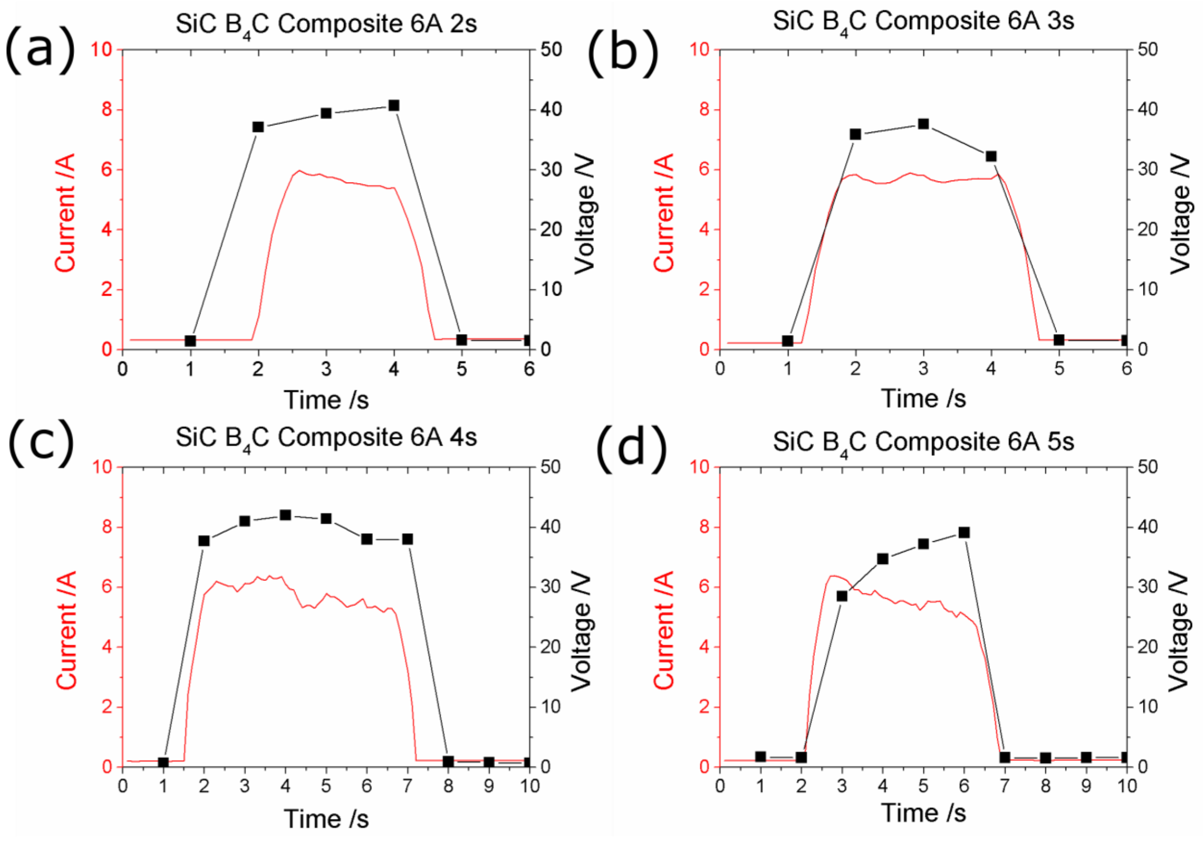

Fig 8s. A comparison of the current and voltage over time for samples SiC 50 wt% B_4C contactless flash sintered at 6A for; (a) 2s: (b) 3s: (c) 4s; and (d) 5s

Temperature estimation

By comparison with the literature values, Zone2/3 of sample CFSed for 3s should have experienced a temperature in the range of 2100-2300°C. For this estimation, the lower temperature limit of 2100°C comes from the fact that is difficult to achieve dense materials in pressure less conditions below this temperature. Similarly above 2300°C the material should have shown evidence of the formation of eutectic phases, similar to those seen in Zone 1 (figure 7s (b)), which were not seen in Figure 7s (b). As a result of this estimation, the Zone 2/3 was heated from 1400°C (preheating temperature) to 2100-2300°C in 3 seconds, corresponding to a heating rate between 14000 and 18000°C/min. In CFS the very rapid densification might be explained by thermal runaway resulting in local conditions very different from those in conventional sintering, possibly greatly improving the sintering kinetics. Other more exotic electric field effects, such as enhanced short range diffusion[6] or electro-plasticity[7] might play a part. Further work is needed to understand the sintering mechanism and to decouple thermal from non-thermal (intrinsic currents effects) effects on the accelerated densification observed here. Due to these and other effects, it is clear that CFS might have great potentials to densify SiC/B_4C composites and other materials in pressure less conditions.

Low current SiC:B_4C

Now we have a clear understanding of the process for the SiC:B_4C composite at 6A we can compare this behaviour to that at a lower current of 3A. By reducing the current one might expect the process would be slower and so more easily controlled, however, the actual process is much more complicated as described below.

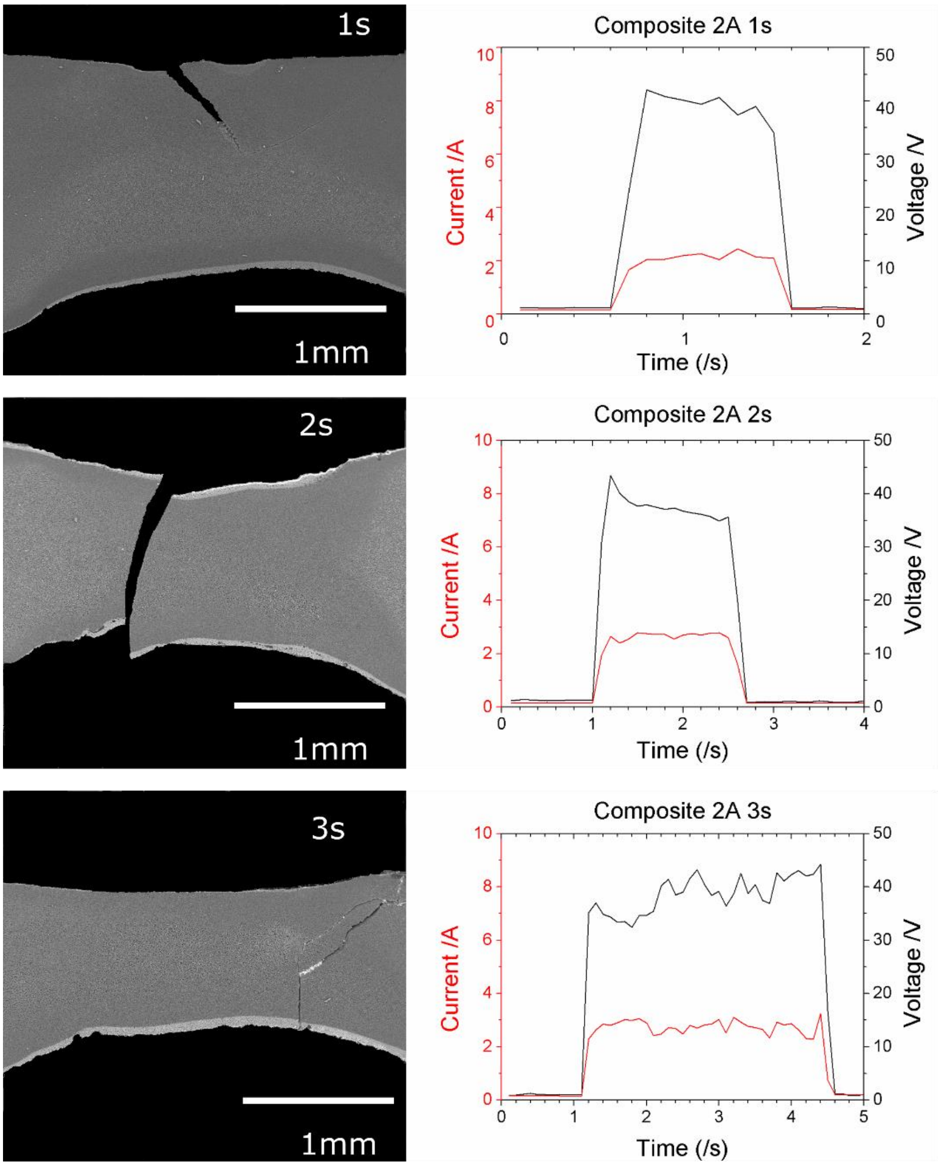

Figure 9s micrographs and electrical data for CFS composite material at a lower current (3A) for a variety of times showing the progression of the process.

While the reduction in thickness seen in Figure 9s is less rapid than in samples prepared at higher currents, as might be expected since a lower current means a lower temperature. However, the sample never achieved full density, and only zone 2 densified, and as time increased the pores in zone 3 actually grew in size. This is hypothesized to be due to the different temperature gradient produced with a lower current. Due to the lower current there is less internal Joule heating and this lead to the sample mostly heating from the surfaces, and

a dense crust formed that sealed in the porosity in the 3rd zone, explaining why it does not densify even at the longest time 3s (actually 3.5s).

Pure B_4C

Pure B_4C is normally sintered at higher temperature than the composite as it does not form a liquid phase (melting point of B_4C is 2700°C). Given this, one might expect the conditions required for CFS to be much harsher than for the composite. However due to the unusual nature of arcs resistance, as mentioned earlier, the actual conditions used were mildest of the materials trailed.

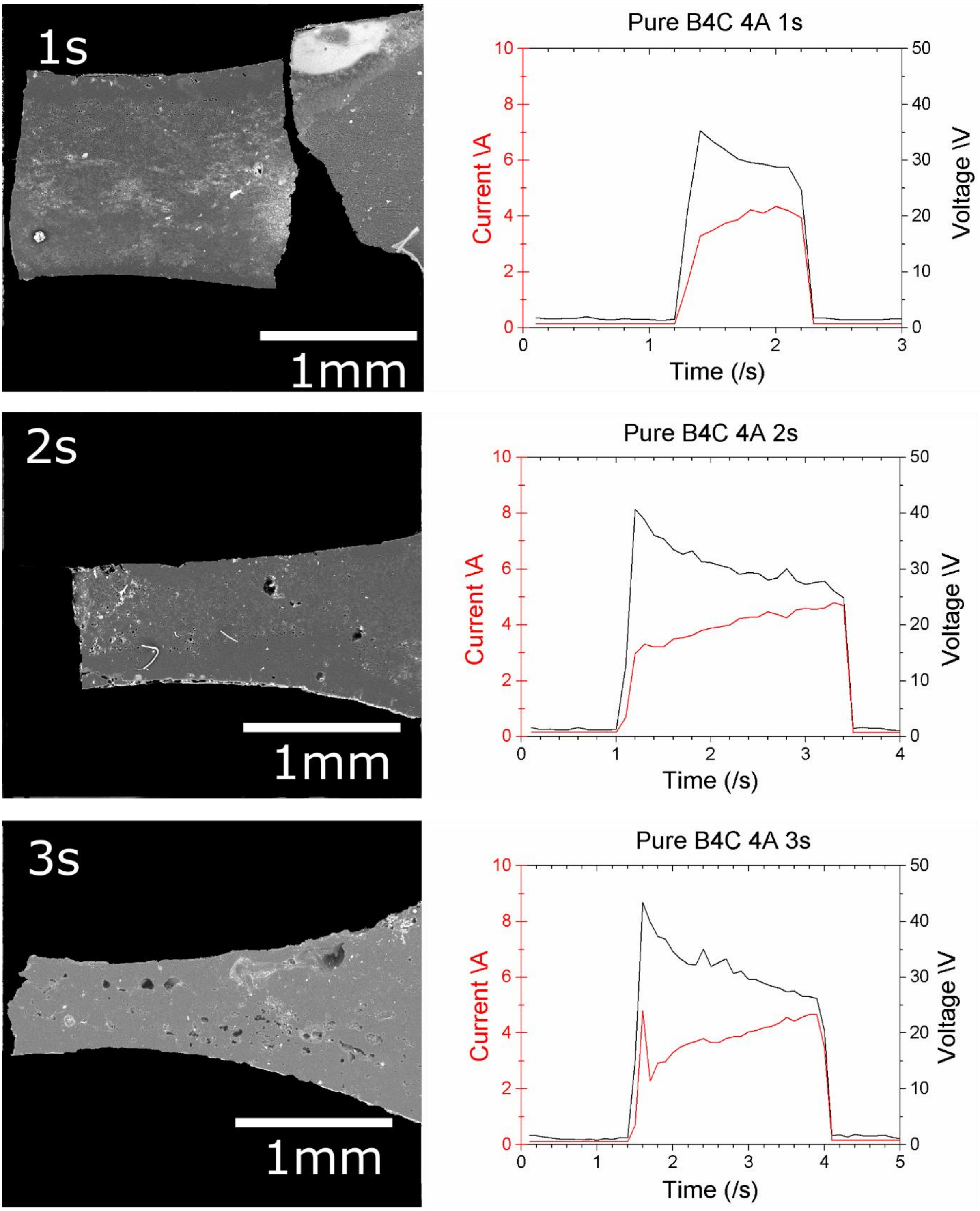

Figure 10s. Micrographs and electrical data for pure B_4C showing the progression of the sintering process.

From the progression of the CFS it is obvious the material is very sensitive to time, and 2s produced the best microstructure, 1s being too short to produce even modest densification and 3s producing bubbles from overheating in zone 3. Also worth noticing is the absence of zone 1 which was very clear in the composite. This is likely due to the increased stability of the surface phase. In the composite, zone 1 is obviously discoloured in the micrograph, due to preferential SiC evaporation leading to a B_4C rich zone (Figure 7s (b)). This did not happen with pure B_4C as the pure material has a much higher melting point, and the surface was not melted by the arcs, making zone 1 and 2 the same. Due to the rapid processing, just an extra half a second of current induced bubbles, The sintered region was also smaller, which might explain how a higher temperature material sintered faster at a similar power to the composite, because of the higher effective power density. A possible reason for the more focused sintering for B_4C is due to the gradient of the temperature vs electrical resistivity curve. A steeper curve would tend to cause the current to focus as the sample heats up. Unfortunately the resistance of the system is not a reliable indicator of the resistance of the sample so this is just conjecture, however, the downward drift in resistance as the sample sinters points towards this.

Incubation period

Given the interesting electrical properties of the pure B_4C a brief investigation was made into the preheating of the sample. For this the separation between the preheating arcs and the sample was increased (distance between electrodes increased to 16mm). With this arrangement the sample should reach a lower stable temperature before the CFS power is applied. The exact temperature reached could not be measured as the recorded value was very sensitive to thermocouple placement, and the thermocouple itself altered the temperature. This change also made the flash sintering very unreliable with most of the samples failing to flash.

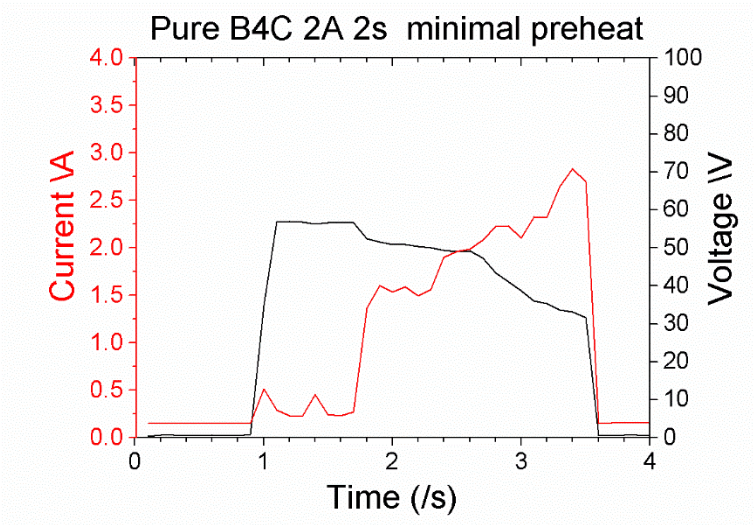

Figure 11s. The electrical data for low preheating pure B_4C, showing incubation time.

A power spike is not visible during CFS as even though the voltage may drop across the sample the voltage drop across the arc is likely to be much higher and dominate. From this we can surmise that we operated well above the flash sintering threshold temperate (for our given voltage) for all our other experiments. This is in good agreement with Richard Todd's work on modelling the incubation time, which shows that for sufficiently high preheating the incubation time tends towards 0 s[8].

Pure SiC

Of the materials and conditions trialled, SiC proved to be the most challenging, and despite our best efforts refused to sinter, but perhaps that is to be expected, for even recent processes like flash and flash SPS have both required substantial pressure to achieve near full density [9,10] and CFS is by its nature is pressureless.

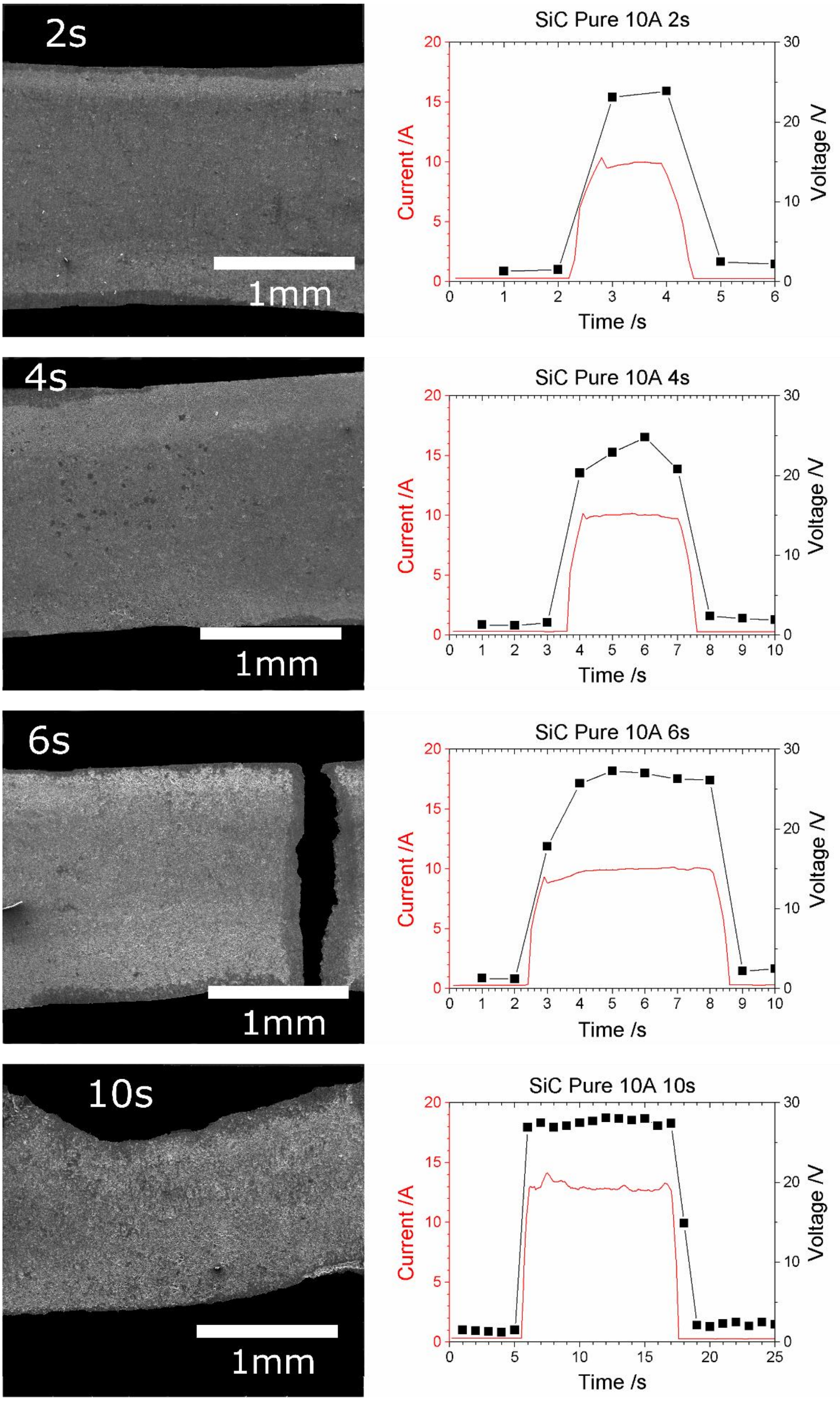

Figure 12s. Micrographs and their accompanying electrical data for SiC samples CFSed at 2, 4 ,6 and 10s.

From the sequence above (Figure 12s), it is obvious that very little densification of SiC took place, as the sample did not thin. A closer inspection showed that the SiC merely sublimed

and redeposited much like during pressureless recrystallization of SiC, a commercial process used to strengthen SiC parts without altering their dimensions. From Figure 12s the sequence of events can be reconstructed. As the sample heated up SiC from the surface started to sublime forming larger pores and an open cell structure in zone 2. When the power was cut the surface cooled and SiC recondensed on the surface leaving a dense coating in zone 1. For longer processing times the sample heated up hot enough for sublimation to occur rapidly in the entire thickness of the sample[3]. Now material was lost to the surroundings as there was no cool region for SiC to redeposit and this lead to a thinning of the sample as seen in the 10s sample.

Summary

From these various investigations it is evident that CFS although a complex process can be understood and optimized for a variety of materials. Great care must be taken when choosing materials and processing conditions, but when operated correctly the process is able to densify material without having to make physical contact with the material. Not only this, the factors that affect the process have been examined in detail, allowing potential further optimization or scale up, which open the door to a new and novel sintering process.

References

1 Ltd, T. *Static arc characteristic*, <http://www.twi-global.com/technical-knowledge/job-knowledge/power-source-characteristics-121/>

2 Secrist, D. Phase Equilibria in the System Boron Carbide-Silicon Carbide. *J. Am. Ceram. Soc.* **47**, 127-130 (1964).

3 Lilov, S. Study of the equilibrium processes in the gas phase during silicon carbide sublimation. *Mater. Sci. Eng., B* **21**, 65-69 (1993).

4 Lankau, V., Martin, H.-P., Hempel-Weber, R., Oeschler, N. & Michaelis, A. Preparation and Thermoelectric Characterization of SiC-B4C Composites. *J. Electron. Mater.* **39**, 1809-1813 (2010).

5 Wurst, J. & Nelson, J. Lineal Intercept Technique for Measuring Grain Size in Two-Phase Polycrystalline Ceramics. *J. Am. Ceram. Soc.* **55**, 109-109 (1972).

6 Olevsky, E. & Froyen, L. Constitutive modeling of spark-plasma sintering of conductive materials. *Scr. Mater.* **55**, 1175-1178 (2006).

7 Francis, J. S. & Raj, R. Flash-Sinterforging of Nanograin Zirconia: Field Assisted Sintering and Superplasticity. *J. Am. Ceram. Soc.* **95**, 138-146 (2012).8 Todd, R., Zapata-Solvas, E., Bonilla, R., Sneddon, T. & Wilshaw, P. Electrical characteristics of flash sintering: thermal runaway of Joule heating. *J. Eur. Ceram. Soc.* **35**, 1865-1877 (2015).

9 Zapata-Solvas, E., Bonilla, S., Wilshaw, P. & Todd, R. Preliminary investigation of flash sintering of SiC. *J. Eur. Ceram. Soc.* **33**, 2811-2816 (2013).

10 Grasso, S. *et al.* Flash Spark Plasma Sintering (FSPS) of α and β SiC. *J. Am. Ceram. Soc.* (2016).
